# Reducing local tension to repair nasal septal deviation and spur

**DOI:** 10.1016/j.bjorl.2024.101464

**Published:** 2024-07-08

**Authors:** Qihang Lin, Xi Lin

**Affiliations:** a The First Affiliated Hospital of Fujian Medical University, Department of Otorhinolaryngology, Head and Neck Surgery, Fuzhou, China; b Binhai Campus of The First Affiliated Hospital, Fujian Medical University, National Regional Medical Center, Department of Otorhinolaryngology, Head and Neck Surgery, Fuzhou, China

**Keywords:** Nasal septal deviation, Endoscopic septoplasty, Local tension, Spur

## Abstract

**Objectives:**

To introduce our method managing nasal septal spurs during endoscopic septoplasty.

**Methods:**

We conducted a prospective study of cases treated with endoscopic septoplasty between March 2022 and June 2023. We innovated a surgical method to reduce the local mucosal tension at the spur by cutting the spur above and below the bony connection, and reducing the chance of mucosal tear and loss during dissection. The authors performed these cases at the First Affiliated Hospital of Fujian Medical University, where 40 surgeries were performed with regular postoperative follow-ups for 6–12 months.

**Results:**

All patients' clinical symptoms improved significantly after surgery. After 2–4 weeks of follow-up, the mucosa could recover to the preoperative state on both sides of the nasal septum.

**Conclusion:**

This surgical method is suitable for most patients with nasal septal deviation, especially those with a spur, which can effectively reduce the chance of nasal septal mucosa tear and accelerate postoperative recovery.

**Level of evidence:**

Ⅳ.

## Highlights

• All patients' clinical symptoms improved significantly after surgery.
• It can reduce the chance of mucosa tear and accelerate postoperative recovery.
• No complications were found.
• It’s especially suitable for the septal deviation with spur.

## Introduction

Nasal septal deviation is a common disease of otolaryngology, which can cause a variety of nose symptoms, such as nasal congestion, nosebleed, headache, vertigo and tinnitus. The traditional surgical method of nasal septal correction was pioneered by Killian (1904), namely submucosal resection of the nasal septum. With the popularization of nasal endoscopy, endoscopic septoplasty has been gradually accepted. Compared with the traditional method, endoscopic septoplasty has many advantages. The operator can see the anatomy, which can accurate operation and directly handle the parts of the septum deviation. It can also be used as a preparation for nasal endoscopic sinus surgery. Surgery trauma and bleeding are far less than traditional surgery, so endoscopic septoplasty has become mainstream at home and abroad.^1^

In nasal septal deviation, the spur is common abnormal nasal septal deviation anatomy, and most of the septum correction surgery injuries occur here. During stripping of the local septum mucosa flap, due to spur deviation, the bone is sharp, the local mucosa is thin, and the mucosa here is easy to rupture, even leading to large mucosal avulsion, missing. It affects wound healing and may cause complications such as nasal septum perforation.

We innovated a surgical method to reduce the local mucosal tension at the spur by cutting the spur above and below the bony connection and reducing the chance of mucosal tear and loss during dissection. This surgical method is suitable for most patients with nasal septal deviation, especially those with a spur, which can effectively reduce the chance of nasal septal mucosa tear and accelerate postoperative recovery. This article shares our experience in managing nasal septal spurs during endoscopic septoplasty. This surgical method has not been previously described in the literature.

## Methods

We conducted a prospective study of cases treated with endoscopic septoplasty between March 2022 and June 2023. The authors performed these cases at the First Affiliated Hospital of Fujian Medical University, where 40 surgeries were performed with regular postoperative follow-ups for 6–12 months. All patients had symptoms of nasal congestion, headache in three patients, nosebleed in one patient, and snoring in nine patients. These symptoms existed all the time.

The inclusion criteria are patients with symptomatic nasal septal deviation. All patients underwent primary septoplasty. To exclude nasal congestion and headache caused by other factors. Our exclusion criteria are patients with severe sinusitis、nasal tumor or previous septoplasty.

The patient was prepared before surgery for standard septal surgery and placed in the supine position. The nasal cavity was injected with tecaine containing epinephrine, 1% lidocaine, and epinephrine 1:100,000 along the subperichondrium of the left nasal septum.

The front end of the nasal septum is 0.5 cm from the cartilage. Killian incision is made from the top to the bottom of the nose. The incision depth is appropriate to open the perichondrium. The perichondrium and the nasal septal cartilage are separated from the front to the back until the spur is exposed.

**Fig. 1:**
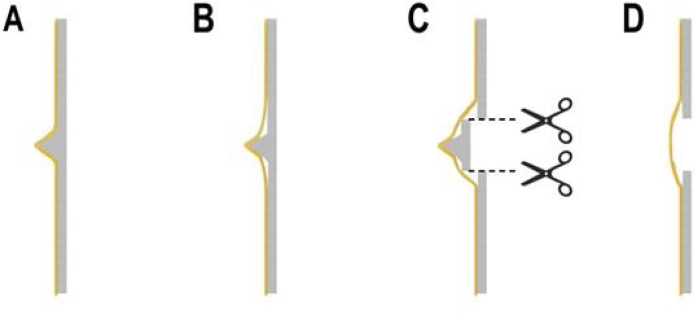
Schematic diagram of nasal septal deviation correction. In the deviated nasal septum, the spur is visible (A). The mucous chondrium and periosteum are separated above and below the spur (B). The bone connection above and below the spur is cut off to reduce the mucosal tension (C). Then the spur and the nasal septum are separated (D).

**Fig. 2:**
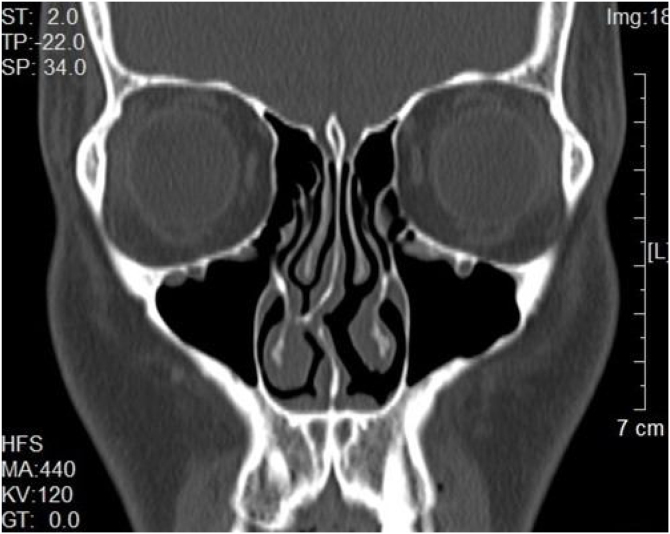
Preoperative CT imaging of the nasal septum with deviation to the right and compression of the ipsilateral inferior turbinate.

**Fig. 3:**
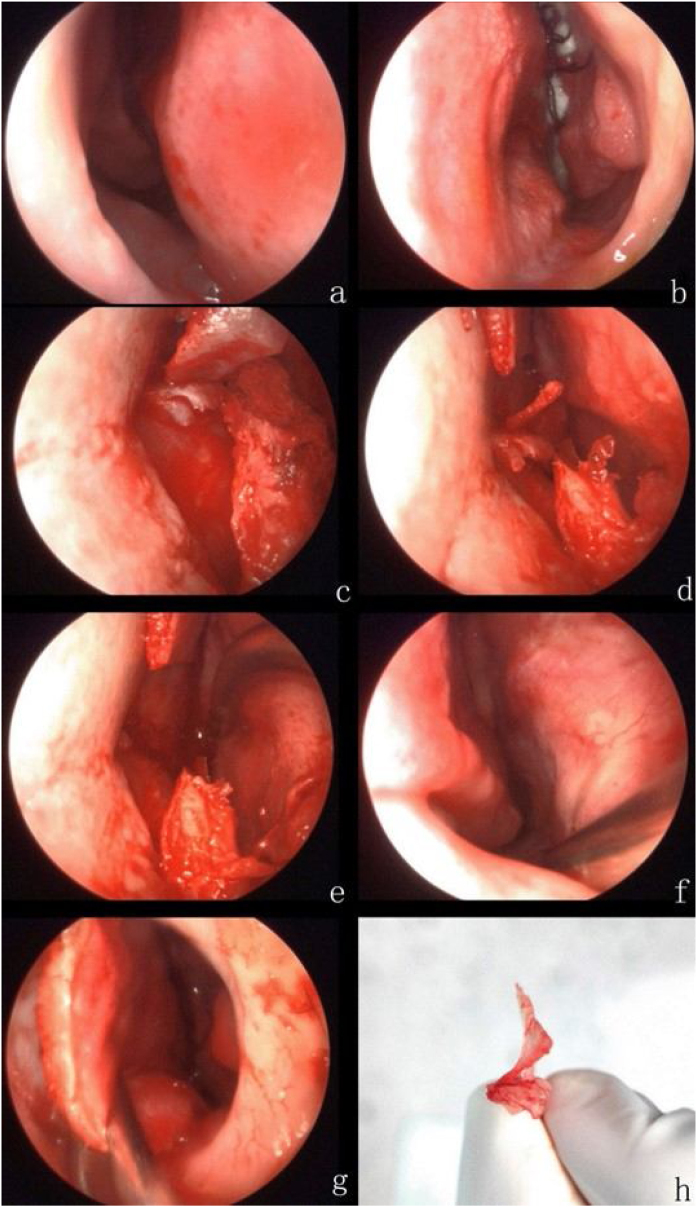
The right nasal cavity before endoscopic septoplasty. The nasal septum deflects to the right, and the horizontal right spur can be seen (a). The left nasal cavity before endoscopic septoplasty (b). The Killian incision is made at the left septum. Then we separate cartilage and mucous cartilage and make a vertical incision between the septal cartilage and bone (c). Separate the superior and inferior bone (d). Remove spur and partial septal bone (e). Examine the right septum mucosa without perforation and mucosal tear (f). Reset the left septum mucosa (g). The spur and nasal septum (h).

**Fig. 4:**
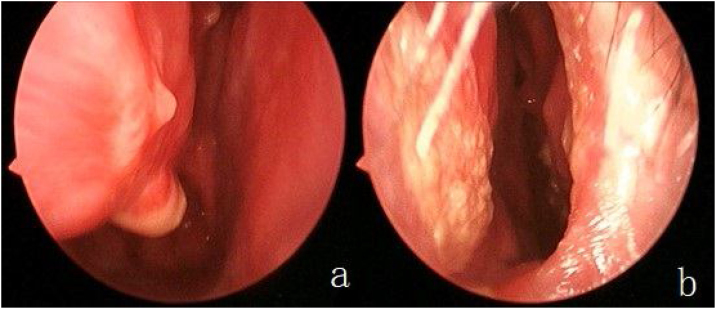
Review of endoscopic septoplasty. The spur was removed in the right nasal cavity, and the mucosa was intact (a). The left nasal incision healed (b).

After exposing the spur, we find the straight part above and below the septum mucous membrane and periosteum. Then we expand its space with a bony nasal septum, release here and cut the bony connection. We separate the crooked spur from the nasal septum cartilage or bone (plow bone, sieve bone vertical plate, or maxillary palate nasal ridge), then remove the mucosa at the spur. The spinous local mucosal tension decreases, and the stripping mucosal tear and missing change are greatly reduced. After that, the nasal septum mucosa was reset and pushed to the midline, stitched with the 4‒0 absorbable suture to fix the nasal septum and make the nasal septum flat and straight (Fig. 1, Fig. 2, Fig. 3, Fig. 4).

In this study, we focused on the improvement of nasal congestion symptoms and the degree of nasal patency, so as to understand the efficacy of the surgery. At the same time, during the follow-up process, we also focused on the complications of surgery, including nosebleed, nasal septal perforation, nasal septal hematoma, and nasal adhesion.

For the evaluation of the patient's condition, we can observe the patency of the nasal cavity under the endoscopic video, and ask the patient whether the symptoms of nasal congestion have improved before and after surgery, so as to judge the changes of the patient's condition.

## Results

This study included 40 cases with the age range of 17–54 years. All patients had improved nasal congestion symptoms, while headache and nosebleed symptoms disappeared, while in patients with snoring, 7 patients still had snoring symptoms. These patients were followed for 6–12 months during the present study period.

In the study, before surgery, we found that the nasal septum narrowed the nasal cavity. Under the 0 ° nasal endoscopic video, the olfactory fissure area of the deviated side and even the middle nasal tract was blocked by the deviated nasal septum, and the patency of the nasal cavity was not good, which affected ventilation. Some of the septal spinous process compression of the ipsilateral turbinate, may cause headache, nosebleed and other symptoms.

Because this method has no large mucosal avulsion or absence, the chance of complications of this operation is very small. In the postoperative nasal endoscopy, we found that the deviated nasal septum had been corrected. Under the 0 ° nasal endoscopy video, the mucosa of the olfactory fissure and the middle nasal tract could be easily observed. At the same time, the spur of the nasal septum was removed, and the turbinate was no longer compressed.

During the postoperative follow-up, two patients appeared nasal adhesion and one patient appeared nasal bleeding, and there are no patients with nasal septal hematoma or perforation so far. The patients with the above complications have been treated by nasal endoscopy, and the complications have been cured. The mucosa matched and healed well after surgery. After 2–4 weeks of follow-up, the mucosa could heal completely, and all recovered to the preoperative state on both sides of the nasal septum.

## Discussion

The nasal septal deviation refers to the septum deviation from the midline or irregular deviation, affecting normal nasal function. It can cause nasal congestion, headache, nosebleed, and other symptoms. At present, it can be observed that there are many types of nasal septal deviation, which can be divided into C-type or S-type deviation, bone spur, and maxillary crest. Because nasal ventilation is guaranteed by the passage at the bottom of the nasal cavity between the nasal septum and the lower nasal tract, the purpose of nasal septoplasty is to correct the anatomical deformity of the nasal septum, reduce the compression of the deviated nasal septum on the inferior turbinate, broaden the airflow channel, and improve the ventilation function of the nasal cavity.^2^

Conventional septoplasty is usually performed with headlights, or headlamp lighting, which is technically challenging because the surgical field of the nasal cavity, especially the posterior segment, is limited. Moreover, the teaching process of surgery becomes very difficult due to limited visualization.

Endoscopic septoplasty is a better alternative to conventional inferior headlight septoplasty. This method provides a more accurate operation, improving the operator’s surgical field, especially in the treating the posterior septum deviation. It not only can evaluate the nasal septum to the inferior nasal turbinate compression and the inferior turbinate compensatory hyperplasia but also can evaluate the width of the nasal canal and olfactory fissure, which provides the operator of the nasal sinus drainage unobstructed assessment. In addition, through endoscopy, the surgeon allows to visually evaluate the nasal tract, avoiding affecting the surgical field because of the distortion of the nasal anatomy. Therefore, during surgery, endoscopic septoplasty allows for a better assessment of improved ventilation function in critical areas of obstruction, such as the nasal flap area. Meanwhile, endoscopic septoplasty is also a valuable teaching tool. Using video displays to demonstrate the steps and techniques of mucosal flap dissection provides important learning opportunities for surgeons and operating room staff compared to traditional headlights. Thus, traditional headlight-based surgery has largely been replaced by endoscopy.^3^

Nasal endoscopic septum surgery has developed rapidly in recent years, while many improved surgical options emerge constantly.^4^, ^5^, ^6^, ^7^, ^8^ However, there is no satisfactory solution for the spur processing of the nasal septum. In most articles, the spur’s septal mucosa during dissection.^3^, ^5^ In our clinical practice, we found that even though the spinous mucosa was treated carefully and slowly, the problem of mucosal rupture and avulsion was still unsolved and unsolved. Some scholars proposed removing the mucosa during the spur process.^9^ However, in the case of the overall deviated nasal septum, it is still necessary to separate the cartilage and the much cartilage. Moreover, if the mucosa behind the spur is not easy to cut, there is still a chance of mucosal avulsion.

The nasal septal spur occurs at the back of the front of the plow bone, the posterior edge of the sift vertical plate and the junction of the septal cartilage. It usually comprises the lateral septal cartilage and the plow bone, often forming sharp processes.^10^ In the posterior end of the septum to one side of the inferior turbinate, the patient, at the same time, can also be complicated with headache, nosebleed and other symptoms.^11^ In septal plastic surgery, the sharp spur is also one of the key points of operator management.

**Fig. 5:**
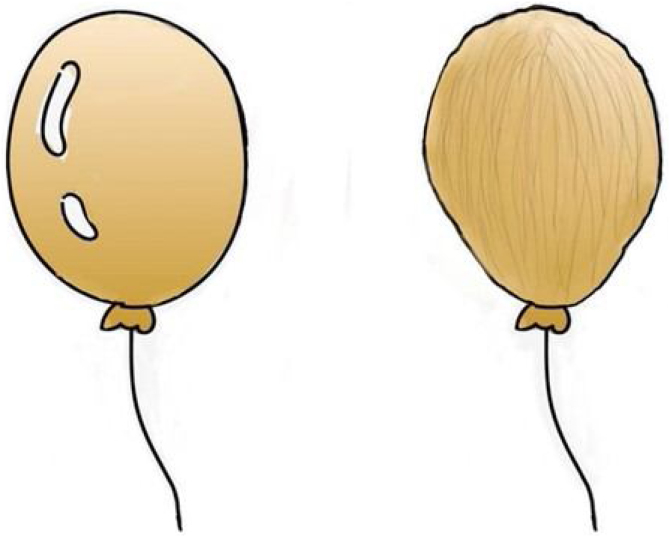
The pressure and tension on the surface of the air-filled balloon is greater than that of the unfilled balloon, and it is more likely to burst.

According to the Laplace formula, the local pressure is correlated to the tension to a centainextent.^12^ In the tight plane, the tension is large, and the local pressure is also large. On the contrary, the tension of the loose plane is small, and the local pressure is relatively small, just like the same size balloon. When applied to our nasal septum surgery, the air-filled balloon is more likely to burst (Fig. 5).

**Fig. 6:**
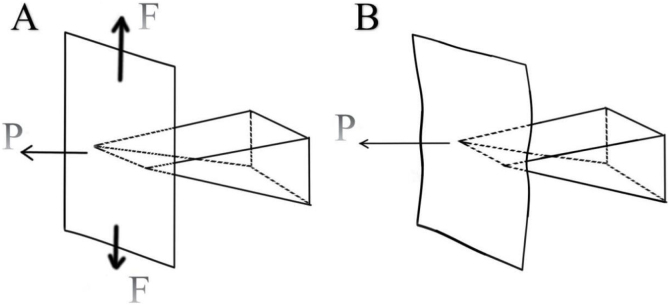
When the mucosa is intact, the tension is large, the mucosa is tight, and the pressure is easy to tear (A). When the upper and lower bones are cut, the mucosal mobility is large, and the tension is reduced, reducing the possibility of tearing (B).

In correcting the spur, the previous literature mentioned the complete separation of the mucosa in the spur process, and then the resection of the spur process. However, in the actual operation process, we found that the local dissection can very easily lead to laceration at the apex mucosa of the spur (Fig. 6).

We presume nasal septum mucosa as a large curvature plane. When the spur exists, the nasal septum mucosa is equivalent to a tight plane. The tension from the surrounding direction and the pressure at the tip are very big. Any operation may make the nasal septum mucosa tear, even large mucosa avulsion and missing, which affects the postoperative septum wound healing. The chance of causing complications increases, such as septum perforation. If we first reduce the local tension during the dissection process and relax the septal mucosa, then there is an opportunity to protect the septal mucosa. Therefore, we have innovated a nasal septal surgery scheme, especially suitable for the spur. That is reducing local tension to repair nasal septal deviation and spur.

In endoscopic septoplasty, the method is safe and effective. Some studies have divided nasal septal deviation into seven types.^13^ This surgical method is better for nasal septal deviation with a unilateral spur process for its correction and mucosal protection effect. This method has an obvious effect and is more effective in protecting the mucosa. The retained mucosa can also cover the wound as soon as possible. Even if there is damage, the residual mucosa is usually in a good position to accelerate the recovery of the nasal septum. Whether this surgical method has significant complications, the nasal septal mucosal flap can return to preoperative within 2–4 weeks.

In conventional septoplasty, if the spur is existed, it is difficult to be directly removed. According to the size of the spurs the Angle of the septal deviation and the proficiency of the operator, the duration of the operation will be extended. Our method reduces the local tension, on the one hand, lessens the impact of spurs and nasal septum deviation. On the other hand, reduces the difficulty of surgery, which is more suitable to beginners' learning.

## Conclusion

Reducing Local tension is very helpful for endoscopic correction of nasal septal deviation, which is suitable for most patients with septal deviation, especially for the septal deviation type with spur. This method can reduce nasal septal mucosa damage, accelerate patient wound healing, and reduce postoperative complications. This method is a safe and effective technical innovation.

## Ethical statement

This surgical method has been widely used in our department, and patients have signed inform consents before surgery. No tissue samples have involved. So the institutional review board (IRB) reviews were waived based on the institutional policy.

## Disclosure statement

No potential conflict of interests by the authors. The authors alone are responsible for the content and writing of the paper.

## Conflicts of interest

There is no actual or potential conflict of interest including any financial, personal, or other relationships with other people or organizations within three years of beginning the submitted work that could inappropriately influence, or be perceived to influence, their work.
